# Evaluation of anticancer properties of a decoction containing *Adenanthera pavonina* L. and *Thespesia populnea* L.

**DOI:** 10.1186/s12906-016-1053-9

**Published:** 2016-02-20

**Authors:** Indeewari K. S. Lindamulage, Preethi Soysa

**Affiliations:** Department of Biochemistry and Molecular Biology, University of Colombo, Colombo, Sri Lanka

**Keywords:** *Adenanthera pavonina*, *Thespesia populnea*, Apoptosis, Traditional decoction, Cytotoxicity

## Abstract

**Background:**

A decoction composed of *Adenanthera pavonina* L. and *Thespesia populnea* L. is currently being used in the treatment of cancer patients.

**Methods:**

Lactate Dehydrogenase (LDH) release, (3-(4, 5-Dimethylthiazol-2-yl)-2, 5-diphenyltetrazolium bromide) MTT, and Sulforhodamine B (SRB) assays were carried out to study cytotoxicity and anti-proliferative activity against the HEp-2 cells, 24 h post-treatment with the decoction.

**Results:**

The mean (± SD) values of EC_50_ were 195.50 (±40.68), 120.02 (±29.82) and 77.06 (±8.80) μg/ml for LDH, MTT, and SRB assays respectively. These results strongly correlate the morphological changes observed in cells treated with the decoction. Induction of apoptosis was visualized by fluorescence microscopy stained with ethidium bromide/acridine orange dye mix. In addition, brine shrimp lethality assay showed an EC_50_ value at a higher concentration (1.96 mg/mL).

**Conclusions:**

These results suggest that the decoction prepared with *Adenanthera pavonina* L. and *Thespesia populnea* L. exhibits anti-proliferative activity and induces apoptosis on the HEp-2 cancer cells but no toxicity against *Artemia salina*.

## Background

Cancer has been an important issue in medicine as it is one of the major causes of death in both the developed and developing countries and is now recognised only to be secondary to myocardial infarction [1]. The discovery of novel anticancer therapeutics from natural sources, mainly from plants of medicinal value remains pivotal. Firstly, the broad spectrum of inherent toxicities associated with contemporary anticancer agents and secondly the acquired resistance to currently available drugs warrants cancer researchers to explore further, the ability of natural products to engage as potential anticancer agents [2]. Traditional medical practitioners have been using crude drug extracts and biologically active compounds, isolated from plant species for a number of years. Some of the prescriptions used in Sri Lanka have been studied for anticancer activity in vitro using cancer cell lines [3, 4]. In our study, we are evaluating the anticancer properties of a complete decoction currently used to cure chronic cancer patients. The decoction used in the present study contains *Adenanthera pavonina* L. and *Thespesia populnea* L. Both of these plants have numerous medicinal applications. Some of them include the anti-diabetic, anti-hyperlipidemic activity [5], antinociceptive, anti-inflammatory effects [6], antibacterial activity [7] and pharmacological effects related to Alzheimer’s disease [8] by *Thespesia populnea* L. Similarly, *Adenanthera pavonina* L. contains anti-inflammatory [9, 10], anti-bacterial [11], anti-hyperglycemic, lipid reducing potential [12] antioxidant and cytotoxicity properties [13]. A decoction prepared in combination with these two plants, *Adenanthera pavonina* L. and *Thespesia populnea* L. has been used for years to cure cancer patients by indigenous practitioners. A previous study carried out in our laboratory has shown that the same decoction possesses strong antioxidant activity in vitro [14]. Hence, the present study was carried out to evaluate the scientific validity of the decoction on anticancer properties.

## Methods

### Materials and equipment

Chemicals were purchased from Sigma Chemicals Co. (P.O. Box 14508, St. Louis, MO 63178 USA) and Fluka (Flukachemie GmbH, CH-9471 Buchs). Lactate Dehydrogenase (LDH) enzyme assay kits were purchased from Roche (Roche Diagnostics GmbH, Germany) and Randox (Randox Laboratories Ltd., Crumlin Co. Antrim, UK). All chemicals used were of analytical grade. The decoction was freeze dried using LFT 600EC freeze dryer equipped with an external pump (Hitachi, Japan). SHIMADZU UV 1601 UV- Visible spectrophotometer (Shimadzu Corporation, Kyoto, Japan) was used to read the absorbance. Cells were observed using Olympus (1X70-S1F2) inverted fluorescence microscope (Olympus Optical Co. Ltd. Japan), and Nikon D700 (105 mm macro lens, film speed 1000 asa, Japan) was used to capture the photographs. Deionised water used in all experiments was obtained from LABCONCO (waterproplus) UV ultra-filtered water system (LABCONCO Corporation, Kansas city, Missouri 64132–2696).

### Plant materials and preparation of the decoction

The phytochemical composition of this decoction was previously determined by our laboratory [14]. The mean ± SD of the total phenols and flavonoids were determined to be 34.14 ± 3.54 *w/w* % gallic acid equivalents and 42.40 ± 0.39 *w/w* % EGCG equivalents respectively. The presence of these phytochemicals may contribute to the apoptotic effects observed. To prepare the decoction, the barks of *Thespesia populnea* L. (Indian tulip tree/Pacific rosewood/Bebaru/Gansuriya) were collected from live plants in Deniyaya (Matara District) and the barks of *Adenanthera pavonina* L. (Red bead tree/Coral tree/Red Sandalwood tree /Madatiya) were obtained from Kotte, Colombo district, Sri Lanka [14]. All plant materials were identified and confirmed by the Department of Botany, Bandaranayake Memorial Ayurveda Research Institute, Nawinna, Colombo, Sri Lanka and voucher specimens are available at the same premises. The barks of *Thespesia populnea* L. (30 g) and *Adenanthera pavonina* L. (30 g) were used in equal proportion to prepare the decoction. The recipe for decoction was provided by Dr. Nimal Jayathilake, Consultant Physician, Bandaranayake Ayurvedic Research Institute, Nawinna, Maharagama. All the plant ingredients were made into smaller pieces and a fine powder was obtained using a clean kitchen blender. The contents were then boiled with 1.6 L of water in a clay pot with a lid. Boiling was allowed to continue until the volume decreased to 200 ml (1/8th of the original volume). The aqueous extracts were subsequently decanted and filtered through a piece of clean cheese cloth (Vijay Auction, Colombo, Sri Lanka). The extract was then centrifuged at 1000 rpm for 5 min. The supernatant was retained and the pellet was solubilised with a minimum quantity of deionised water, sonicated and centrifuged at 2500 rpm for 8 min. Both supernatants were pooled and then freeze dried. The freeze dried samples were stored at −20 °C in sterile tubes until further use. Three individually prepared decoctions were made according to the above procedure.

### Cell line

HEp-2 cell line originally obtained from Medical Research Institute, Sri Lanka, which was preserved and maintained in the Department of Biochemistry and Molecular Biology, was used for all cytotoxic experiments.

### Cell culture and preparation of the cells for cytotoxicity

Cells were cultured in Eagle’s Minimum Essential Medium (EMEM) supplemented with 10 % fetal bovine serum (FBS), MEM non-essential (1 %), L-glutamine (3 %), 50 IU/ml penicillin and 50 μg/ml streptomycin. The pH of the growth media were adjusted to physiological pH (7.4) using 7.7 % sodium bicarbonate. Cells were maintained at 37 °C in a 5 % carbon dioxide (CO_2_). The cells were seeded in a 24-well plate (2 × 10^5^) and cultured overnight. Confluent monolayer was treated with different concentrations of the decoction at 37 °C for 24 h in humidified CO_2_ incubator. In all experiments, a negative control without the decoction and a positive control with camptothecin (5 mM) were used.

### MTT assay

MTT (3-(4, 5-Dimethylthiazol-2-yl)-2,5-diphenyltetrazolium bromide) assay is a semi-automated colorimetric assay based on the principle that the mitochondria of live cells reduce the tetrazolium salt, MTT to blue formazan compounds [15]. The cells were cultured overnight on 24-well plates as described above and treated with different concentrations (50–500 μg/ml) of the decoction for 24 h at 37 °C in a humidified CO_2_ incubator. The negative control without the plant extract was used and the final volume of each well was adjusted to 1 ml with growth media. After 24 h, the supernatant was subsequently replaced with regular media (1 ml) followed by the addition of MTT (5 mg/ml in PBS). The cells were incubated at 37 °C for 4 h and the media was carefully removed. The formazan crystals were then dissolved in acidified isopropanol (0.05 M HCl in IPA) by mixing well with pipette tip. The absorbance of the resulting solution was measured at 570 nm. Acidified IPA was used to zero the spectrophotometer. The percentage viability was calculated as given below:

$$ \%\ \mathrm{Cell}\ \mathrm{Viability}=\left(\mathrm{Absorbance}\ \mathrm{of}\ \mathrm{treated}\ \mathrm{cells}\div \mathrm{Absorbance}\ \mathrm{of}\ \mathrm{untreated}\ \mathrm{cells}\right)\times 100 $$

The net absorbance from the wells of untreated cells was taken as 100 % viable. The experiment was carried out in triplicates.

### Lactate dehydrogenase (LDH) activity

The changes in the membrane permeability can be detected by the leakage of enzymes such as lactate dehydrogenase. The cells were treated with the decoction at different concentrations (50 – 500 μg/ml) for 24 h. The culture supernatants (200 μl) and cell lysates (200 μl) were tested for the presence of LDH enzyme, using the LDH detection kit.

$$ \%\  Cytotoxicity=\left(1-LDH\  activity\  in\  the\  supernatant\div Total\ LDH\  activity\right) \times 100 $$

The experiment was performed in triplicates.

### Sulforhodamine B assay

The Sulforhodamine B (SRB) assay, first described by Skehan and colleagues (1990) is based on the ability of the SRB dye to bind electrostatically to the basic amino acid residues [16]. The monolayer of cells was treated at different concentrations (40–160 μg/ml) of the decoction and cultured overnight in a humidified CO_2_ incubator. The medium was then removed completely. The adherent cells were treated with trichloroacetic acid (10 %; 500 μl) to fix the cellular protein and the plate was incubated at 4 °C for at least 1 h prior to the SRB assay. The plate was then washed with five washing cycles using deionised water and dried completely. A volume of 500 μl of SRB (0.4 %; in 1 % acetic acid) was added to each well and allowed to stain for 30 min. The excess stain was removed and the plate was subjected to five washing cycles again to remove unbound dye using 1 % acetic acid (vol/vol). After air-drying, the protein bound dye was solubilised with Tris base (10 mM; 500 μl) and the plates were shaken for at least 30 min. The absorbance was then recorded at 564 nm using Tris base as blank. The percentage viability was calculated as given below:

$$ \%\ \mathrm{Cell}\ \mathrm{Viability}=\left(\mathrm{Absorbance}\ \mathrm{of}\ \mathrm{treated}\ \mathrm{cells}\div \mathrm{Absorbance}\ \mathrm{of}\ \mathrm{untreated}\ \mathrm{cells}\right)\times 100 $$

The experiment was carried out in triplicates.

### Morphological determination

Cells (2 × 10^5^ cells per well), were seeded in 24-well plates and exposed to the decoction at two different concentrations (50 and 100 μg/ml) for 24 h as described earlier. The morphological changes of cells were observed using Olympus (1X70-S1F2) inverted fluorescence microscope at 100X magnification and photographed using Nikon D700 camera. A negative control (untreated cells) and a positive control (Camptothecin: 5 mM) were also analysed for comparison.

### Ethidium Bromide/Acridine Orange Staining (EB/AO staining)

Differential uptake of dyes that bind to DNA is technique that is used to determine the mode of cell death, especially the difference between apoptosis and necrosis [17]. Most of the live cells are permeable to acridine orange (AO) and hence the nuclei of AO-stained cells appear green. In contrary, ethidium bromide (EB), which stains red, enters the cell only when there is a loss in the cell membrane integrity. Thus live cells exhibit green nuclei, early apoptotic cells bright green nuclei with fragmented chromatin and late apoptotic cells have condensed and fragmented orange chromatin. However, the necrotic cells will possess a normal orange/red nucleus with no aberrant changes [18].

Induction of apoptosis by the decoction was investigated with AO/EB dye staining as described by Ribble et al. [18]. The cells (2 × 10^5^) were treated with two different concentrations (50 and 150 μg/ml) of the decoction and cultured overnight in a humidified CO_2_ incubator at 37 °C. The media was transferred to 15 ml tubes. The rest of the adherent cells were detached with Trypsin-EDTA (1 ml) and incubated at 37 °C for 2 min. The media and the detached cells from the same sample were pooled together and centrifuged at 1,000 rpm for 5 min. Cell pellets were then re-suspended in 25 μl cold PBS and 2 μl EB/AO dye mix (EB/AO dye mix contained 100 μg/ml of each dye). Stained cell suspensions (10 μl) were visualized using an Olympus (1X70-S1F2) inverted fluorescence microscope at 100X magnification and photographed using Nikon D700 camera.

### Brine shrimp lethality assay

Brine shrimp lethality bioassay was carried out to investigate the general cytotoxicity of the decoction [19]. Brine shrimps (*Artemia salina*) were hatched using brine shrimp eggs in a petri dish (diameter – 85 mm) filled with 12 ml of filter-sterilized sea water (pH 8.5) for a period of 24 h under aeration. Ten nauplii were drawn through a pipette and placed in 24-well plates (final volume 2 ml) at different concentrations of the decoction. The plate was maintained at room temperature for 24 h under aeration and the surviving larvae were counted. Experiments were conducted along with a negative control. The percentage lethality was calculated from the mean survival larvae of extracts treated with the decoction and control. The experiment was carried out in triplicates.

$$ \%\ \mathrm{Lethality}=\left(\mathrm{Mean}\ \mathrm{survival}\ \mathrm{larvae}\div \mathrm{Mean}\ \mathrm{larvae}\ \mathrm{in}\ \mathrm{untreated}\ \mathrm{control}\right)\times 100 $$

### Calculations and statistics

A minimum of three independent experiments were carried out unless otherwise specified. Linear regression analysis were carried out using Microsoft Excel. Calibration curves of the standards were considered as linear if R^2^ > 0.99. Linear segment of the sigmoid curves of the dose response curves between percentage cell viability and log concentration were used to determine the EC_50_ values.

## Results

### MTT assay

**Fig. 1:**
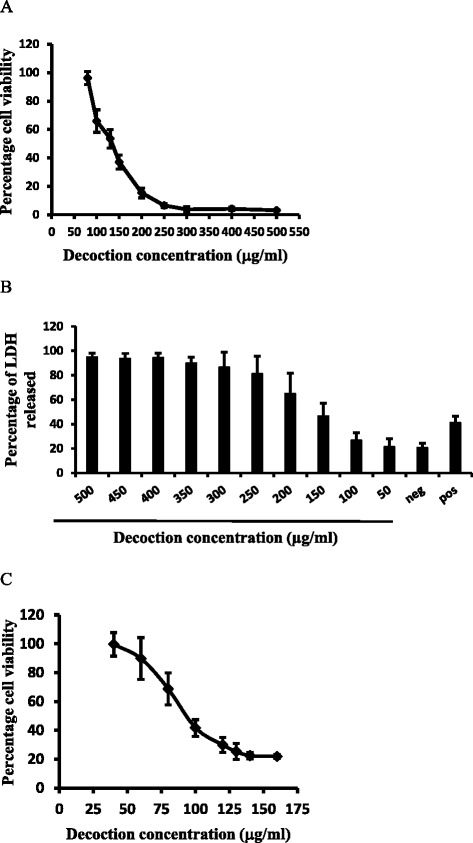
Cytotoxicity of HEp-2 cells induced by the decoction as determined by different cytotoxicity assays. **a** MTT assay was used to determine the percentage cell viability as described in the text. **b** The percentage LDH released after 24 h of treatment with the decoction. Hep-2 cells were treated with different concentrations of the decoction for a period of 24 h. Percentage LDH released was used to construct a dose response curve and the linear segment of this curve was used to determine EC_50_ value. ‘Neg’ and ‘Pos’ indicates untreated and 5.0 mM camptothecin-treated samples respectively. **c** Cytotoxicity of HEp-2 cells induced by the decoction as determined by SRB assay. Dose response curve for cell viability for decoction prepared with *Adenanthera pavonina* L. and *Thespesia populnea* L was used to determine the EC_50_ value using the linear segment of the curve. The results are presented as mean ± SD of at least three independent experiments

The cell viability was determined by MTT reduction assay after 24 h treatment with decoction as described earlier. A dose–response curve for the percentage of viable cells was plotted against concentrations of the extract. Maximum growth inhibition was shown at concentrations over 300 μg/ml, however, the cell viability remained unchanged and the decoction was not effective in cytotoxicity at concentrations <80 μg/ml. The EC_50_ value obtained was 120.02 ± 29.82 μg/ml (Fig. 1a).

### LDH activity

LDH release into the medium and subsequent reduction in NADH concentration was used as an index of the integrity of cell membranes and on this basis, percentage LDH released was calculated as described previously. A dose dependent increase of LDH release was observed with the decoction with an EC_50_ value of 195.50 ± 40.68 μg/ml (Fig. 1b). A maximum inhibition of 80 % was observed at concentrations over 350 μg/ml of the decoction. The LDH release for negative and camptothecin (positive control) were 21 and 41 % respectively.

### Sulforhodamine B (SRB) assay

The effect of the decoction on HEp-2 cell survival was determined by the SRB assay as previously described. A dose-dependent inhibition was observed with an inhibition of 78.54 % at concentration of 160 μg/ml. The EC_50_ value for the decoction was observed at 77.06 ± 8.80 μg/ml (Fig. 1c).

### Cell morphology studies and Ethidium Bromide/Acridine Orange Staining (EB/AO staining)

**Fig. 2:**
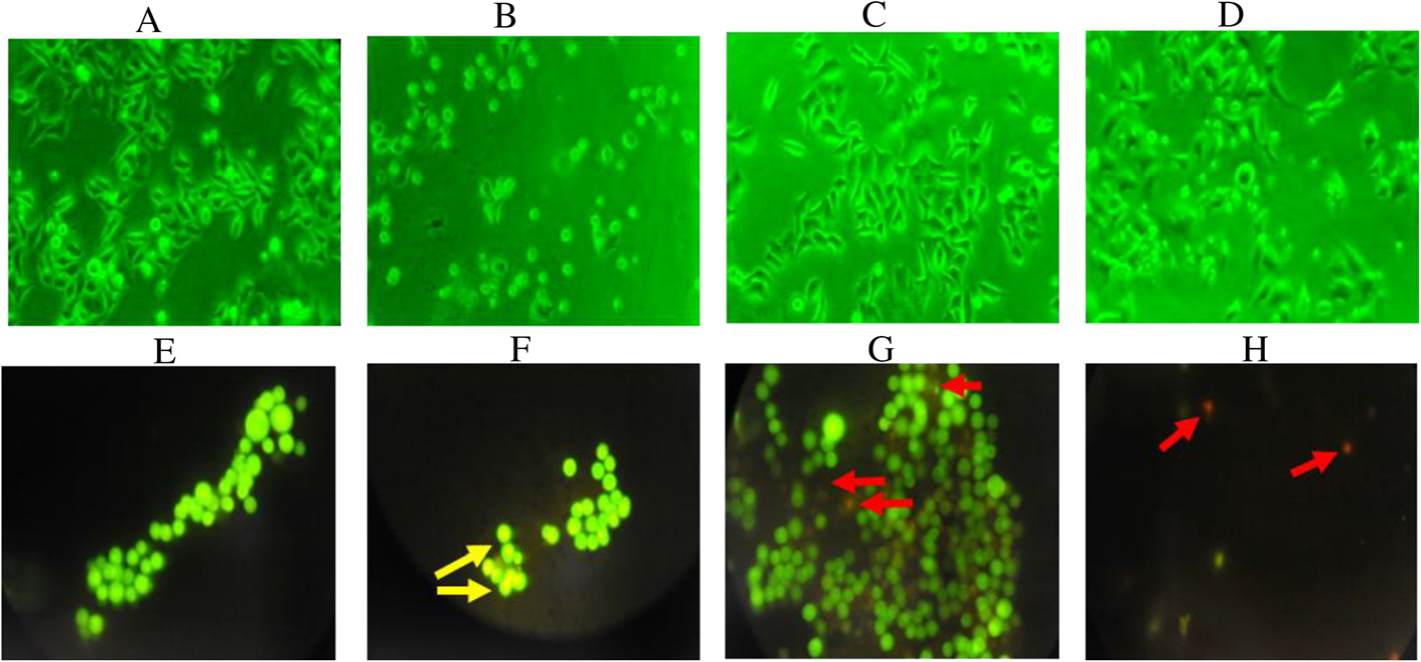
Images of HEp-2 cells on treatment with/without the decoction by light and fluorescence microscopy. (*Top panel*) Light micrographs (Phase contrast) of cultured HEp-2 cells after 24 h of incubation. **a** Untreated control cells **b** Cells incubated with camptothecin (5 mM; 25 μl) as positive control. **c** and **d** cells incubated with 50 and 100 μg/ml of the decoction respectively. (*Bottom panel*) Images of cells after treating with acridine orange-ethidium bromide staining. Untreated cells show a uniform green fluorescence **e**; cells treated with 50 μg/ml decoction appeared green with bright green nuclei indicating nuclear fragmentation and early apoptotic cells **f**; with 150 μg/ml of decoction **g** and camptothecin (5 mM; 25 μl) as the positive control **h** show late apoptotic cells by the orange red appearance due to the incorporation of both ethidium bromide and acridine orange. Yellow and red arrows indicate early and late apoptotic cells respectively. (Magnification 100X)

The cytotoxic effects of the decoction on HEp-2 cells were analyzed using an inverted fluorescence microscope as depicted in the Fig. 2, top panel. The figures show the potential of the decoction in inducing apoptosis in the HEp-2 cells by demonstrating the morphological changes characteristic to apoptosis such as membrane blebbing, cell shrinkage, nuclear and cytoplasmic condensation and formation of apoptotic bodies as compared to the negative control. Apoptotic and necrotic cells were further investigated using fluorescence microscopy and double staining with ethidium bromide and acridine orange (Fig. 2-bottom panel). Live cells displayed a uniformly green fluorescence which had regular, round-shaped nuclei in the absence of decoction (Fig. 2e). The early apoptotic cells were observed at a concentration of 50 μg/ml, which the cells were still green in color with bright green dots in their nuclei corresponding to nuclear fragmentation (Fig. 2f). The late apoptotic cells were visible at concentration of 150 μg/ml of the decoction and in positive control, camptothecin (5 mM) which had stained orange attributed to incorporation of both ethidium bromide and acridine orange (Fig. 2g & h).

### Brine shrimp lethality assay

**Fig. 3:**
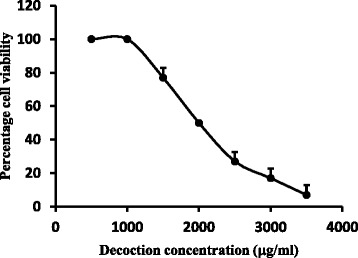
Effect of different concentrations of the decoction on the viability of the brine shrimps. The linear segment of the curve was used to calculate the EC_50_ values by linear regression analysis. The results are presented as mean ± SD of three independent experiments

Brine shrimp lethality is the simplest bioassay useful for screening large number of extracts in the drug discovery process. Preliminary experiments carried out at a concentration range of 50 – 800 μg/ml did not show any toxic effect on brine shrimp assay. However, the number of live brine shrimps decreased with a concentration >1.5 mg/ml with an EC_50_ of 1.96 ± 44.84 mg/ml (Fig. 3).

## Discussion

Complementary and alternative medicine (CAM) is very popular in the contemporary world and is widely adopted around the globe [20]. A decoction, containing barks of *Thespesia populnea* and *Adenanthera pavonina* is used in the treatment of cancer by traditional physicians in Sri Lanka. Lyophilized samples of this extract were studied for its phenolic and antioxidant activity and the findings were previously published [14]. The results of this initial study clearly demonstrated that the potent antioxidant activity of the decoction is justified by its high phenolic constituents and flavonoids. Therefore, we initiated to evaluate the cytotoxic properties using a range of assays to provide evidence of potent anticancer abilities of the decoction.

In this study, the anti-proliferation and induction of apoptosis by the decoction were studied using MTT, LDH and SRB assays with HEp-2 cell line. It was found that the decoction significantly inhibited the proliferation of HEp-2 cells after incubation of the cells for a period of 24 h and the results showed a concentration-dependent decrease in the percentage cell viability of the HEp-2 cell line.

The observation of apoptotic cells at higher concentrations suggest that the effect of the decoction is cytocidal at least at higher concentrations. We also feel that the cytocidal or cytostatic phenotype mostly depends on the concentration used and the incubation time. When we use a concentration close to the EC_50_ of the drug (150 μg/ml), the apoptotic signal appears to be stronger that when the concentration of the decoction was used three times lesser than the EC_50_ (50 μg/ml), suggesting that the effect of the decoction was dose-dependent. The two concentrations of the decoction, we have included in the manuscript, provides two clear phenotypes of early and late apoptosis incurred by the decoction.

Previous studies conducted by Boonsri et al. (2008) evaluated the cytotoxic properties of *Thespesia populnea* [21]*.* In their study, the heartwood and wood were separately extracted from *T. populnea* in dichloromethane and these extracts were shown to induce a potent cytotoxic ability against different cancer cell lines, including MCF-7, HeLa, HT-29, and KB cells. In addition, Johnson et al. (1999) extracted four quinones, namely mansonone-D, mansonone-H, thespone and thespesone from the heartwood of *T. Populnea* and evaluated their cytotoxicity in MCF-7 breast cancer cells [22]. Mansonone-D and Thespone showed enhanced cytotoxic effects than the other two quinones, mainly owing to generation of superoxide anions, which the authors speculate to be responsible for the cell killing effect. Masuda et al. (2002) evaluated the cytotoxic activity of leaf extracts in sea shore plants in the subtropical regions of Japan via flow cytometry using ethidium bromide and annexin V-FITC as fluorescent probes. One of these plants was *T. Populnea* and the leaf extracts exhibited potent cytotoxic effect against human leukemic cell line, K562 [23]. It is interesting to observe the presence of several natural occurring cytotoxic compounds in *T. populnea* and one of compounds, most elaborately studied is Gossypol [24]. Gossypol is present usually in members of the Malvaceae family, including cotton plant (*Gossypium* species) and in *Thespesia populnea*. Gossypol has been shown to exhibit potent anti-proliferation ability against a range of human cancer cells, including ovarian, endometrial, colon, cervical, lung, colon, leukemia and melanoma [14, 15, 17–22, 25-33, 36]. More interestingly, gossypol also showed cytotoxic property against cell lines resistant to vinblastine, adriamycin and cisplatin [29]. Further studies involving clinical trials showed minimal adverse effects, however the response rates of the receiving patients were low [34–36]. Hence additional studies need to be performed to explore either a new extract or a synergistic combination with an additional compound.

Studies were performed with the barks of *A. pavonina* in respect to their anti-inflammatory abilities [37]. However, recent reports have also demonstrated the cytotoxic potential of the ethanolic seed extracts of *A. pavonina.* Ferreira et al. (2011) reported that, after 72 h of treatment with ethanolic seed extracts of *A. pavonina* (50 μg/mL), there was a 30.8 ± 5.2, 23.7 ± 3.2, 4.5 ± 2.4, and 1.2 ± 13.2 % proliferation inhibition for HCT-8, SF-295, MDA MB-435 and HL-60 cells respectively [38]. However, studies on cytotoxic potential of the bark of this plant have been explored only to a minimal extent. Hence, this is the first instance where the cytotoxic ability of a decoction composed of barks of *Thespesia populnea* and *Adenanthera pavonina* is described. The decoction displayed cytotoxic effects against the HEp-2 cell line, however, the toxic doses for brine shrimp larvae are in the range 10–100 times higher in comparison to cell culture methods. The brine shrimp lethality assay has been widely used to screen toxicity and the level of toxicity of pesticides, dental materials, crude plant extracts and fractions, secondary metabolites and nanoparticles [39, 40]. Meyer (1982) has reported that extracts obtained from natural products which have LC_50_ ≤ 1.0 mg/mL are known to possess toxic effects [19]. The present study shows an EC_50_ value of 1.96 mg/mL for brine shrimp assay, whereas for studies on anti-proliferation of cancer cells, the value ranges from 77 to 195 μg/ml proving that no such toxic effects exists on *Artemia salina*.. However the toxicity studies against a normal mammalian cell line is still remain to be established.

## Conclusions

The results obtained from the present study shows that the decoction containing *Adenanthera pavonina* L. and *Thespesia populnea* L. possesses potent anti-proliferative and cytotoxic activities (Table 1). The claim by traditional healers that the decoction containing *Adenanthera pavonina* L. and *Thespesia populnea* L. is partially validated in the present study by identifying its aqueous extract to contain apoptotic activity. If some of the compounds are structurally identified and characterized, they may be candidates for further anticancer drug development.

**Table 1:** Summary of the cytotoxic activities of the decoction composed of *Thespesia populnea* L. and *Adenanthera pavonina* L.

EC_50_ (*n* = 3)				
	Brine Shrimp	LDH	MTT	SRB
	(mg/ml)	(μg/ml)	(μg/ml)	(μg/ml)
Decoction	1.96 ± 44.84	195.50 ± 40.68	120.02 ± 29.82	77.06 ± 8.80
Percentage growth inhibition at 5.0 mM				
Camptothecin	ND	41.39 ± 4.92	50.50 ± 6.02	45.06 ± 7.05

*ND* Not determined

## Abbreviations

CAM: Complementary and alternative medicine
CO_2_: Carbon dioxide
EB/AO: Ethidium bromide/acridine orange
EMEM: Eagle’s Minimum Essential Medium
FBS: Fetal bovine serum
HCl: Hydrochloric acid
IPA: Isopropylalcohol
LDH: Lactate dehydrogenase
MTT: (3-(4, 5-Dimethylthiazol-2-yl)-2, 5-diphenyltetrazolium bromide)
SRB: Sulforhodamine B
